# Structure-Based Insights into Stefin-Mediated Targeting of Fowlerpain-1: Towards Novel Therapeutics for *Naegleria fowleri* Infections

**DOI:** 10.3390/ph18111606

**Published:** 2025-10-23

**Authors:** Pablo A. Madero-Ayala, Rosa E. Mares-Alejandre, Patricia L. A. Muñoz-Muñoz, Samuel G. Meléndez-López, Marco A. Ramos-Ibarra

**Affiliations:** 1 Science and Engineering Graduate Program, Autonomous University of Baja California, Tijuana 22390, Mexico; pablo.madero@uabc.edu.mx; 2 Biotechnology and Biosciences Research Group, School of Chemical Sciences and Engineering, Autonomous University of Baja California, Tijuana 22390, Mexico; rmares@uabc.edu.mx (R.E.M.-A.); lilian.munoz.munoz@uabc.edu.mx (P.L.A.M.-M.); samuelmelendez@uabc.edu.mx (S.G.M.-L.)

**Keywords:** AlphaFold2-Multimer, MM-PBSA, PockDrug, papain-like cysteine protease, Fowlerpain-1, cathepsin L homolog, stefin A, brain-eating amoeba

## Abstract

**Background/Objectives**: *Naegleria fowleri* is a free-living protozoan that causes primary amoebic meningoencephalitis, a rapidly progressing central nervous system infection with high mortality rates and limited treatment options. Targeting virulence-associated proteins is essential for effective drug development. Fowlerpain-1 (FWP1), a papain-like cysteine protease (CP) implicated in extracellular matrix degradation and host–cell cytotoxicity, has been investigated as a therapeutic target. This study aimed to evaluate the FWP1 pocket geometry and stefin binding using an integrated in silico structural biology approach. **Methods**: A computational pipeline was used, including AlphaFold2-Multimer modeling of FWP1–stefin complexes, 20-ns molecular dynamics simulations under NPT conditions for conformational sampling, and molecular mechanics Poisson–Boltzmann surface area free energy calculations. Three natural CP inhibitors (stefins) were investigated. Structural stability was assessed using root mean square deviations, and binding profiles were characterized using protein–protein interaction analysis. **Results**: Stable FWP1–stefin interaction interfaces were predicted, with human stefin A showing favorable binding free energy. Two conserved motifs (PG and QVVAG) were identified as critical mediators of active-site recognition. Druggability analysis revealed a concave pocket with both hydrophobic and polar characteristics, consistent with a high-affinity ligand-binding site. **Conclusions**: This computational study supports a structural hypothesis for selective FWP1 inhibition and identifies stefins as promising scaffolds for developing structure-guided protease-targeted therapeutics against *N. fowleri*.

## 1. Introduction

Proteases are enzymes that hydrolyze peptide bonds, and are essential for protein turnover and other critical biological processes. Based on their active-site chemistry and catalytic mechanisms, they are classified as cysteine proteases, serine proteases, threonine proteases, aspartic proteases, glutamic proteases, and metalloproteases. Understanding their structure–function relationships is essential for developing targeted therapeutics for many human diseases [1,2,3,4,5].

Cysteine protease (CP) enzymes use a conserved catalytic triad composed of cysteine (nucleophile), histidine (proton acceptor), and asparagine (geometry stabilizer) [4,6,7]. They are synthetized as inactive zymogens and activated by the removal of a propeptide [6,8]. These proteases have been widely investigated as therapeutic targets for inhibitor development because of their central roles in diverse pathological processes, including host–pathogen interactions in infectious diseases [9,10,11,12].

The Cystatin superfamily includes a diverse group of competitive, reversible, and tight-binding inhibitors that regulate polypeptide degradation [13,14]. According to the MEROPS classification, most endogenous inhibitors of papain-like proteases belong to the I25 family, which is divided into I25A (stefins), I25B (cystatins), and 12C (kininogens) [4,15]. In particular, stefins inhibit papain-like proteases by inserting a wedge-shaped loop into the catalytic cleft, thereby preventing substrate access [16,17].

*Naegleria fowleri*, the “brain-eating” amoeba, is a thermophilic, free-living protozoan found in freshwater environments that can tolerate temperatures of up to 45 °C. In humans, it causes primary amoebic meningoencephalitis (PAM), a rare but devastating infection of the central nervous system (CNS), with a high mortality rate. Global analyses have reported a case fatality rate of approximately 73%. In contrast, historical datasets suggest a rate of over 95%, highlighting both the lethality of PAM and the likelihood of underreporting or diagnostic bias [18,19,20,21]. Infections typically occur when individuals inadvertently inhale amoebae while swimming in contaminated waters. Once in the nasal cavity, the pathogen traverses the cribriform plate and infiltrates the brain, causing severe CNS damage [18,19,22]. PAM progresses exceptionally rapidly, often causing death within 3–7 days of symptom onset, and its overlap with other CNS infections complicates timely diagnosis and treatment [22,23,24,25]. Furthermore, the limited efficacy of currently available treatments for *N. fowleri* highlights the urgent need for novel therapeutics that can act effectively within a narrow clinical window [18,26,27].

Advances in genomics, transcriptomics, and proteomics, combined with the current availability of sequence–structure–function data obtained through bioinformatics analysis, have accelerated the discovery of novel proteins and refined the known virulence factors of *N. fowleri* [28,29,30,31,32]. Proteases are crucial factors that contribute to tissue invasion and immune evasion. However, no clinically validated inhibitors are currently available, encouraging the prioritization of parasite-specific proteases as potential targets [33,34]. Fowlerpain-1 (FWP1), a papain-like CP homologous to cathepsin L (CTSL), was initially described as a secreted 30-kDa enzyme capable of degrading the extracellular matrix and inducing cytopathic effects in mammalian cells. Inhibitor-based studies further demonstrated that blocking proteolytic activity reduces host cell damage [35].

Given that FWP1 is a validated component of the *N. fowleri* pathogenic machinery, it represents a promising therapeutic target for PAM [35,36]. However, its inhibition has not been characterized at the atomic level, and its selectivity relative to human cathepsins remains unclear. This lack of knowledge highlights the need for both computational and experimental approaches to define the structural and functional basis of FWP1 inhibition. Stefins offer a helpful framework, as human and amoebic homologs have shown high-affinity interactions with papain-like proteases in other systems [36,37,38].

No previous research has modeled FWP1 in 1:1 complexes with stefins. In this study, a structure-based computational pipeline integrating AlphaFold2-Multimer predictions, molecular dynamics (MD) simulations, and binding free energy calculations was used to characterize the interactions between FWP1 and stefins in heterodimeric complexes. In silico analysis evaluated interface stability, quantified binding energetics, assessed druggability, and identified key structural determinants of high-affinity inhibition. These findings provide novel insights into stefin-mediated inhibition of FWP1, suggest testable hypotheses for future experimental validation, and support the rational design of selective FWP1 inhibitors as therapeutics for PAM treatment.

## 2. Results

### 2.1. Fowlerpain-1 Displays Papain-like Structural Features

**Figure 1:**
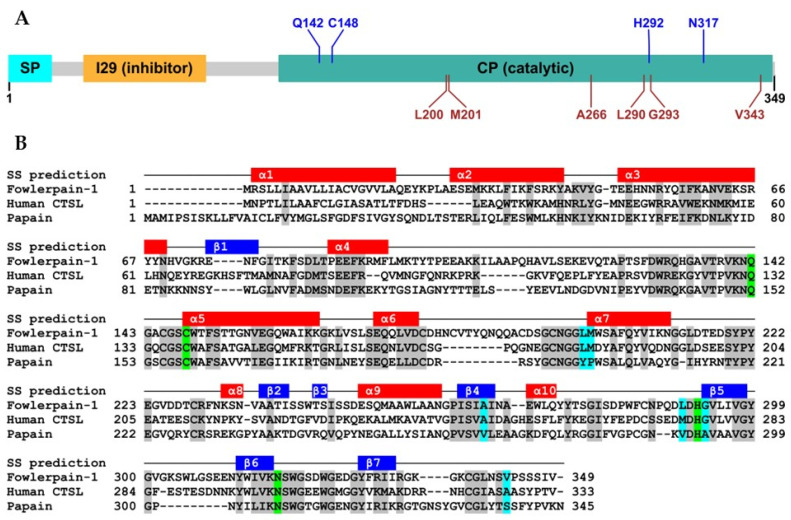
Primary and secondary structures of FWP1. (**A**) Illustration of protein architecture: signal peptide (cyan), propeptide region (orange), and protease domain (turquoise). The locations of the residues involved in proteolysis (blue) and those defining the substrate-specific S2 subsite (brown) are indicated. (**B**) Multiple sequence alignment of FWP1, human cathepsin L (CTSL), and papain. Conserved residues are shaded grey, but those involved in catalysis (green) and substrate specificity (cyan) are shaded differently. The predicted secondary structures (SS) are displayed as red (α-helices) and blue (β-sheets) rectangles above the sequences.

FWP1 exhibited the hallmark primary and secondary structural features of the CP C1A subfamily (also known as the papain-like family). It is synthetized as a 39 kDa pre-proenzyme and matures into a 26–29 kDa active enzyme, consistent with a previous report [35]. Moreover, it shares features with functional orthologs [8]: a signal peptide (Met1–Ala20), a propeptide domain (Phe35–Phe90; E-value = 8.57 × 10^−13^), which belongs to the I29 protease-inhibitor family (https://www.ebi.ac.uk/merops/inhibitors/, accessed on 4 March 2025), and a catalytic (CP) domain (Ala124–Ile348; E-value = 1.43 × 10^−109^) that is highly similar to that found in papain-like enzymes (Figure 1A). Homology analysis confirmed that it shares significant identity with human CTSL (38%) and papain (34%). Secondary structure predictions confirmed a conserved framework consistent with shared sequences and enzymatic functions (Figure 1B).

The catalytic domain of FWP1 contains a conserved triad (Cys148, His292, and Asn317) within three canonical sequence patterns associated with eukaryotic CP enzymes (^142^QGACGSCWTFST^153^, ^290^LDHGVLIVGYG^300^, and ^312^YWIVKNSWGSDWGEDGYFRI^331^). A conserved glutamine residue (Gln142), which is crucial for stabilizing enzyme-substrate intermediates during the catalytic reaction [6], as well as various residues that contribute to defining the substrate-specific S2 subsite were also identified (Leu200, Met201, Ala266, Leu290, Gly293, and Val343).

The AlphaFold-generated three-dimensional (3D) model provided additional information on the catalytic domain of FWP1. The predicted local distance difference test (pLDDT) yielded scores >90 for most regions (Supplementary Figure S1), indicating a highly confident conformation [39,40]. Additionally, the Ramachandran plot confirmed structural reliability, with 87% and 13% of residues in the favored and allowed regions, respectively, whereas the estimated Z-score (−6.64) was within the expected range for reliable structural quality (Supplementary Figure S2). These complementary analyses further validated the stereochemical plausibility of the generated conformations.

**Figure 2:**
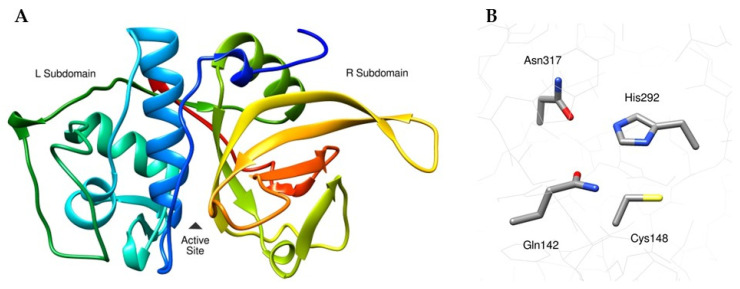
Tertiary structure of the catalytic domain of FWP1. (**A**) Ribbon representation of the best 3D model, rainbow-colored (blue to red) according to the amino-carboxy progression. The location of the active site is indicated by an arrowhead. (**B**) Arrangement of active site residues involved in proteolysis (sticks with default element colors).

The predicted 3D model displayed a classic papain-like fold (Figure 2) consisting of two well-defined subdomains (designated L and R) separated by a deep active site cleft [6,41,42]. Four bonds were identified in the structure. Three of these bonds correlate with the conserved stabilizing framework found in functional papain-like proteases [42]: Cys145↔Cys196 and Cys179↔Cys229 in the L subdomain and Cys285↔Cys338 in the R subdomain. An additional disulfide bond, Cys183↔Cys192, was identified in the L subdomain 16–24 Å from the active site cleft and was structurally compatible with the papain-like protease fold. Its remote location suggests that it contributes to global stability while maintaining the flexibility required for catalysis.

### 2.2. Fowlerpain-1 Exhibits a Reliable Active-Site Cleft

3D modeling predicted a native-like fold for FWP1, which was validated by high local confidence scores and a Ramachandran distribution with all torsion angles (ϕ, ψ) in favorable conformations. This tertiary structure revealed a reliable concave active site cleft bordered by catalytic and substrate recognition residues, providing a consistent framework for ligand-binding site analyses (Figure 2).

Quantitative pocket analysis using CASTpFold identified a primary active-site cavity (Pocket ID 1), with calculated dimensions of 75.2 Å^3^ for volume and 82.3 Å^2^ for the solvent-accessible surface. These geometrical values indicate an accessible cleft with favorable physicochemical properties that can support a wide range of non-covalent interactions. The overall features of the FWP1 active site cleft (Figure 2A) were consistent with those described for other papain-like proteases, such as human CTSL, establishing its potential as a promising site for studying stefin-mediated inhibition [41].

### 2.3. Stefins Form Stable Complexes with FWP1

**Figure 3:**
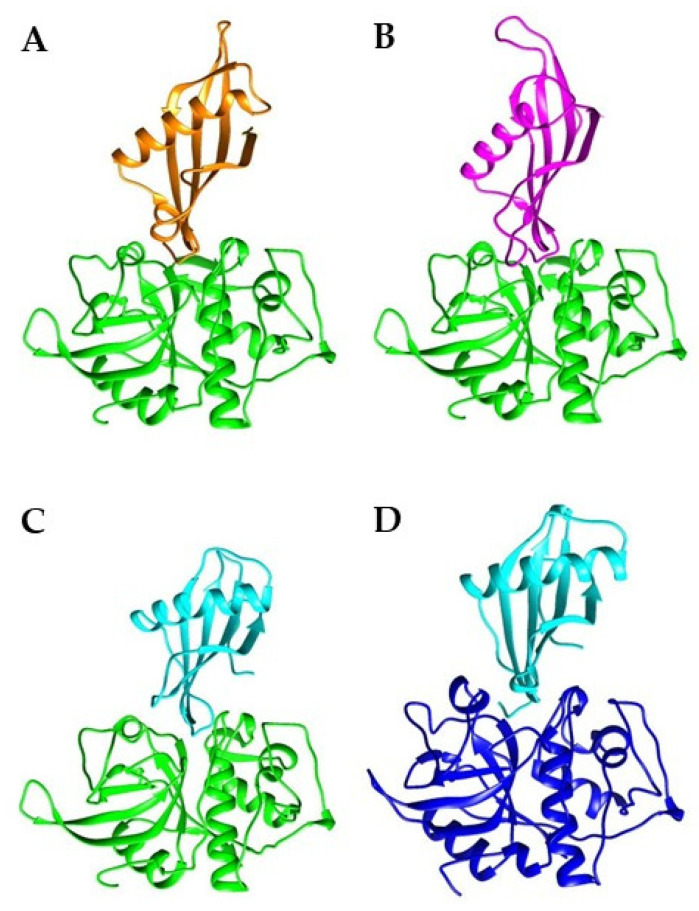
Heterodimeric complexes of FWP1 and stefins. Ribbon representation of the best 3D model for each complex: (**A**) FWP1-FSTF, (**B**) FWP1-NfCPI, and (**C**) FWP1-STFA. The CTSL-STFA complex is also shown in (**D**). Colors: FWP1, green; FSTF, orange; NfCPI, magenta; CTSL, blue; STFA, cyan.

Interactions between FWP1 and three different stefins, fowlerstefin (FSTF), *N. fowleri* cysteine protease inhibitor (NfCPI), and human stefin A (STFA), were investigated using heterodimeric modeling and MD simulations to evaluate interface stability and binding energetics. This computational approach helped determine whether stefins could provide structural insights to guide the design of PAM inhibitors. All heterodimeric complexes generated by AlphaFold2-Multimer exhibited high-confidence pLDDT scores (>90; Supplementary Figure S3). Structural investigation of the resulting FWP1–stefin complexes revealed well-defined interfaces (Figure 3A–C), whereas the human CTSL-STFA complex (control) displayed comparable confidence levels (Figure 3D), supporting the reliability of this prediction approach.

**Figure 4:**
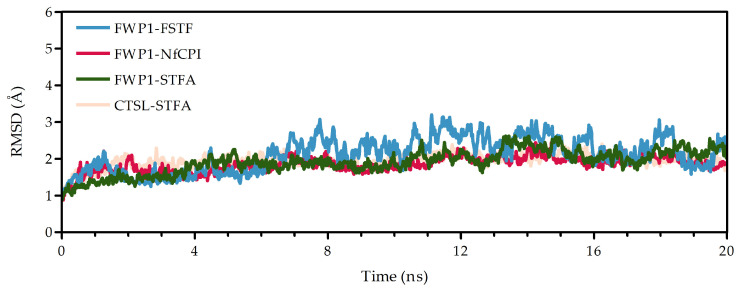
RMSD (root mean square deviation) of the MD trajectory of protease–stefin complexes (upper left, abbreviations as specified in Figure 3). RMSD plots from 20 ns of the simulated heterodimer dynamics (color-coded).

MD simulations confirmed that all three complexes, FWP1-FSTF, FWP1-STFA, and FWP1-NfCPI, were structurally stable throughout the trajectory, with backbone RMSD values maintained at 2.0 ± 0.2 Å (Figure 4), demonstrating equilibrium and persistent interfaces. Reliably, molecular mechanics Poisson–Boltzmann surface area (MM-PBSA) free energy (ΔG) calculations revealed favorable binding across all complexes, with the following ranking: FWP1-FSTF > FWP1-STFA > FWP1-NfCPI (Table 1). Notably, ΔG values were used as a comparative measure to provide relative rankings rather than absolute affinities. Importantly, the control (CTSL-STFA) reproduced the experimental values of ΔG and K_d_), further validating the computational pipeline and supporting the reliability of the FWP1–stefin complex predictions.

**Figure 5:**
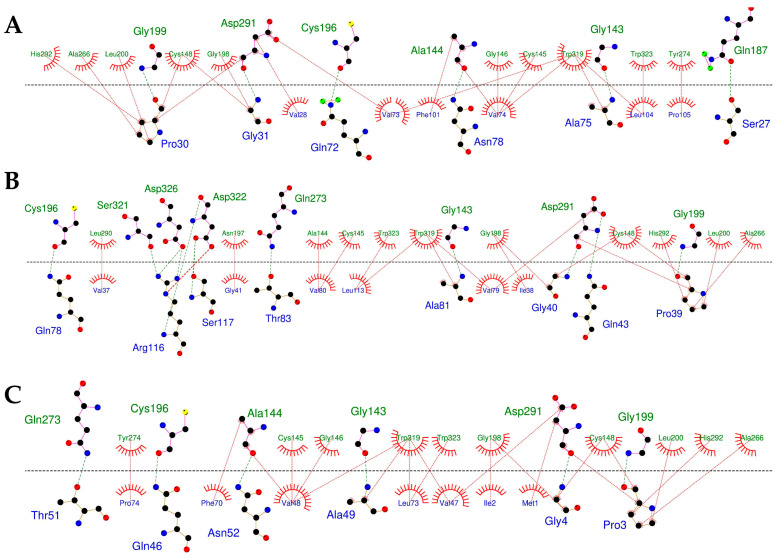
Protein–protein interactions at the interface of FWP1–stefin complexes. DIMPLOT diagram for each complex: (**A**) FWP1-FSTF, (**B**) FWP1-NfCPI, and (**C**) FWP1-STFA, where FWP1 residues are shown in green and those of the stefins are shown in blue. All representations were rendered using the default settings [44,45].

Supplementary interface profiling using DIMPLOT revealed conserved non-covalent networks across all FWP1–stefin complexes, which were primarily stabilized by hydrophobic contacts and hydrogen bonds (Figure 5). Hydrophobic contacts were defined as interactions between non-polar side-chain atoms within a distance ≤4 Å, whereas hydrogen bonds were identified using standard geometric criteria, specifically donor–acceptor distances ≤3.5 Å and donor–hydrogen–acceptor angles ≥135°. The hydrophobic/hydrophilic ratio (HHR), calculated from the MD-derived clusters, helped distinguish stronger binders from weaker binders (for comparative purposes). FWP1-FSTF and FWP1-STFA exhibited higher HHR values (~3.2), indicating stronger binding than that of FWP1-NfCPI (~1.3), which is consistent with their ΔG values (Table 1). This correlation highlights the role of hydrophobic packing in stabilizing complexes and identifies human (STFA) and amoebic (FSTF) stefins as promising scaffolds for FWP1 inhibition.

**Figure 6:**
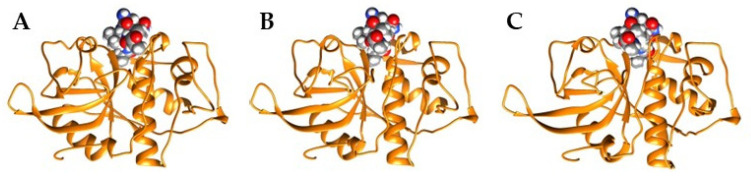
Structural interactions between conserved motifs in stefins (PG and QVVAG) and the FWP1 active site cleft. Illustrations of specific motif-mediated contacts from FSTF (**A**), NfCPI (**B**), and STFA (**C**). Ligand atoms were rendered as spheres with default settings for color and volume, and FWP1 is shown as a bright orange ribbon.

Residue-level analysis identified two conserved stefin motifs, PG and QVVAG, as critical anchors for FWP1 binding (Supplementary Figure S4). The PG motif (Pro30–Gly31 in FSTF, Pro39–Gly40 in NfCPI, and Pro3–Gly4 in STFA) establishes strong hydrophobic contacts with the catalytic and substrate-recognition residues of FWP1, including Cys148, Gly198, Leu200, Ala266, Asp291, and His292. The QVVAG motif (Gln72–Gly76 in FSTF, Gln78–Gly82 in NfCPI, and Gln46–Gly50 in STFA) formed additional interactions with Ala144, Cys145, Gly146, Asp291, and Trp319 (Supplementary Table S1). The persistence analysis revealed that both motifs maintained hydrogen bond occupancy of 25–30% with average distances of 2.8–3.0 Å throughout the simulations. These features highlight the significance of these motifs in stabilizing stefin-binding. Remarkably, the contact between the PG motif and catalytic residues supports a competitive inhibition mechanism in which stefins engage the active site of FWP1, thereby blocking the catalytic cleft (Figure 6).

### 2.4. The Stefin-Binding Region of FWP1 Has Druggable Features

**Figure 7:**
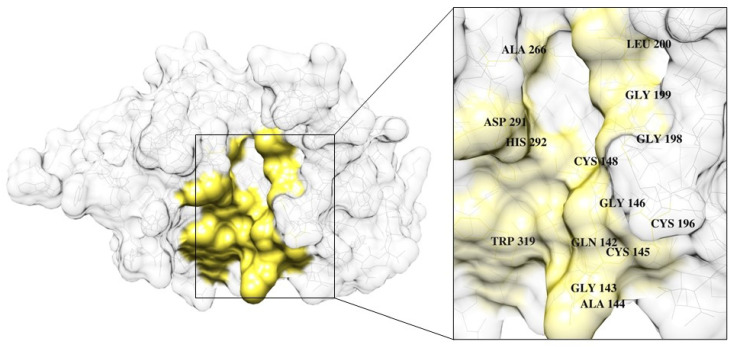
Potential druggable region of FWP1. Left: Surface representation of FWP1 (gray), highlighting the ligand-interaction pocket (yellow). Right: Magnified view (2×) showing conserved residues that define the potential druggable region, including the catalytic dyad (Cys148, His292), disulfide bond cysteines (Cys145, Cys196), substrate recognition sites (Leu200, Ala266), and surrounding anchors contributing to the pocket surface (Gln142, Gly143, Ala144, Gly146, Gly198, Gly199, Asp291, Trp319). A semi-transparent rendering was applied to improve label visualization.

Ligand-binding site analysis identified a conserved stefin-interacting pocket on the FWP1 protease surface, comprising 13 residues that consistently engaged in binding. Complementary hotspot mapping using FTMap revealed that Gln142, Cys148, Gly199, Asp291, and His292 were anchor residues that mediated inhibitor–target interactions (Supplementary Figure S5). These hotspots coincided with the conserved non-covalent contacts observed in the MD simulations, reinforcing their role as structural determinants of selective inhibition. The binding interface displayed the hallmark characteristics of druggable protein surfaces [46], with a concave topology enriched with hydrophobic and polar regions. In addition to the catalytic Cys148 and His292, residues involved in substrate recognition (Leu200 and Ala266) and disulfide bonding (Cys145 and Cys196) were also identified. Remarkably, Gln142 (which was not previously recognized as essential for stefin binding) emerged as an additional anchor residue (Figure 7). These results define a reliable group of residues that can serve as focal points for the rational design of FWP1-selective inhibitors and novel PAM-targeted therapeutics.

A comparative analysis of FWP1 and its closest structural human homolog, CTSL, highlighted the importance of residue-level selectivity in the rational development of specific inhibitors. ConSurf mapping revealed the conservation of the catalytic dyad (Cys148/His292 in FWP1; Cys138/His276 in CTSL) and structural stabilizers (Gln142, Cys145, and Gly199 in FWP1; Gln132, Cys135, and Gly181 in CTSL), reflecting the shared evolutionary and functional constraints within the catalytic core (Supplementary Table S2). Although Leu200 in FWP1 and Leu182 in CTSL are synonymous residues, their local microenvironments differ, suggesting possible effects on substrate accommodation and inhibitor binding. In contrast, the divergence at Ala144 in FWP1 (Gln134 in CTSL) represents a true local chemical variation near the catalytic cleft that can be exploited for a selective structure-based drug design.

A quantitative druggability assessment confirmed the therapeutic potential of this site. The FWP1 catalytic cleft displayed a druggability probability of 0.82 ± 0.02, estimated using PockDrug and DoGSiteScorer (above the 0.5 threshold), which was supported by favorable geometric features (volume: 75.2 Å^3^; surface: 82.3 Å^2^). In contrast, the CTSL pocket displayed similar dimensions (volume: 50.0 Å^3^; surface: 99.6 Å^2^) and comparable druggability probability (0.86 ± 0.03), highlighting the challenge of achieving selectivity based solely on global geometry. Nonetheless, interface-level differences in the substrate-binding subsites provide fine-grained structural features that can be exploited as anchors for designing selective inhibitors. These computational predictions suggest that FWP1 is a druggable target, highlighting the importance of optimizing strategies that focus on local environmental differences rather than the overall geometry of the target.

## 3. Discussion

### 3.1. Structural and Therapeutic Implications of FWP1

Structural analyses confirmed that FWP1 is a secreted papain-like protease, characterized by a conserved catalytic triad (Cys148, His292, and Asn317) and a well-defined substrate-binding cleft. In addition to the three canonical disulfide bonds found in typical papain-like enzymes [42], FWP1 contains another (Cys183↔Cys192), which is presumed to enhance global stability by constraining loop flexibility and decreasing conformational entropy while preserving the plasticity required for enzyme activity [47]. Pocket analysis further demonstrated a geometrically accessible active-site cleft with favorable physicochemical properties, thereby supporting the rationale for the design of substrate-based inhibitors [48]. MD simulations revealed that FWP1 formed stable complexes with stefins: FSTF, NfCPI, and STFA. The binding free energies calculated from the heterodimer dynamics were aligned with the strength of the interactions at the protein–protein interface. Remarkably, the data showed that the binding affinity was correlated with the degree of hydrophobic packing, with FSTF and STFA exhibiting stronger binding affinities than NfCPI. Additional analysis at the residue level highlighted two conserved motifs, PG and QVVAG, which are known to anchor elements of stefins. The PG motif interacts with Cys148 and His292 in the catalytic core, supporting a competitive inhibition mechanism in which stefins block the active site cleft and prevent substrate binding through steric hindrance [38,49,50].

The structural characterization of FWP1 extends its relevance beyond biological settings, highlighting its potential as a therapeutic target. Previous studies have provided compelling evidence that small-molecule CP inhibitors effectively suppress the virulence of *N. fowleri* [35]. This finding reinforces the potential of targeting FWP1 for the development of novel therapies. Comparative conservation mapping against human CTSL, which shares 38% sequence identity with FWP1, revealed a significant similarity. However, specific divergent residues, such as Ala144, provide potential anchor sites for the binding of selective inhibitors. These observations suggest that the successful structure-based design of FWP1-specific inhibitors requires optimization to maximize their selectivity and minimize the risk of off-target effects in vivo.

### 3.2. Stefin-Based Inhibition: Endogenous Against Therapeutic

The application of stefin-based inhibition must be carefully assessed, considering the endogenous stefin repertoire of *N. fowleri*. Despite their intrinsic capability to inhibit CP enzymes, FSTF and NfCPI have functionally diverged to enhance amoebae virulence and survival. FSTF promotes pathogenesis by inducing the production of pro-inflammatory cytokines, thereby exacerbating brain tissue damage during infection [36]. Similarly, NfCPI is selectively expressed during the encystation phase, facilitating cyst formation and ensuring parasite survival in hostile environments [51]. Therefore, these operative adaptations restrict their suitability for direct therapeutic exploitation and their use as structural scaffolds in drug development.

Human STFA is a promising biopharmaceutical candidate for PAM treatment because of its strong predicted binding affinity and structural compatibility with the FWP1 protease. As an endogenous human protein with effective inhibitory capacity, STFA offers an alternative therapeutic profile that distinguishes it from other candidate molecules [52]. While small-molecule inhibitors have shown potential in suppressing *N. fowleri* virulence [35], STFA-based therapeutics offer complementary advantages: high target specificity, reduced off-target toxicity, and lower propensity for resistance. The endogenous nature of this protein further supports its suitability for therapeutic use because it is less likely to trigger adverse immune responses than non-human proteins or synthetic compounds [52,53,54]. Mechanistically, computational studies have predicted that STFA acts by competitively binding to the catalytic cleft, potentially disrupting FWP1-driven proteolytic processes linked to tissue invasion, host-cell cytotoxicity, and immune evasion during *N. fowleri* infections [35,55]. The predicted binding mode also suggests that STFA exhibits a slow dissociation rate from the FWP1 protease, favoring sustained inhibition and prolonged therapeutic effects over the course of PAM progression.

Beyond the therapeutic potential of human STFA, the conserved PG and QVVAG motifs within stefins provide a robust framework for rational structure-based design of peptidomimetics. These motifs provide valuable platforms for engineering synthetic inhibitors that replicate the fundamental binding interactions observed in endogenous and therapeutic stefins [17,30,50,56]. Peptidomimetics can be engineered to preserve essential contacts by introducing targeted modifications that improve both stability and drug-like properties. This approach represents a feasible and significant alternative for developing more effective and safer FWP1-specific inhibitors [14].

### 3.3. Translational Challenges: Blood–Brain Barrier Penetration

One of the most significant translational complications for any stefin-based or protein therapeutic agent against PAM is achieving sufficient penetration across the blood–brain barrier (BBB) to reach effective CNS concentrations [57,58,59]. This is clinically challenging given the rapid progression of PAM, which demands prompt and sustained drug delivery to brain tissue [60]. The limited ability of current treatments, including amphotericin B, to cross the BBB often results in subtherapeutic levels in the cerebrospinal fluid (CSF), contributing to the high mortality rate [61,62]. In this situation, STFA-based inhibition could be employed as an adjunctive therapy to current protocols or as an alternative approach in cases of drug resistance [63,64].

Several advanced and promising drug-delivery strategies have been developed to overcome BBB restriction: (1) intranasal administration, which enables direct nose-to-brain transport through olfactory and trigeminal nerve pathways [65,66]; (2) receptor-mediated transcytosis, which exploits transferrin or insulin receptors that are abundantly expressed in brain endothelial cells [67,68,69]; and (3) nanocarrier systems, such as PEGylated liposomes, polymeric nanoparticles, and engineered exosomes, which can enhance BBB penetration while protecting the cargo from enzymatic degradation [70,71,72,73]. To establish whether STFA-based treatment can be translated, preclinical studies should include pharmacokinetic analyses in appropriate animal models, emphasizing the quantification of CSF/plasma concentration ratios, brain tissue distribution, and persistence of therapeutic levels after administration via different routes [74,75]. Other pharmacological factors must also be considered for successful translation, such as protein stability in biological fluids (i.e., serum and CSF), plasma half-life, susceptibility to degradation, and risk of immunogenicity after repeated administration [76,77,78].

### 3.4. Computational Limitations and Experimental Validation

While the computational predictions generated in this study provide a solid starting point for the development of FWP1 inhibitors, it is worth noting that there are several methodological limitations inherent to these approaches. Although AlphaFold2-Multimer is generally reliable for modeling protein–protein interactions, a minor risk of inaccuracies in interface geometry and side-chain packing persists, particularly for complexes that lack close structural homologs in the training data [79,80,81]. Additionally, the relatively short MD simulations performed in this study, which are sufficient for evaluating the stability of the binding modes, restrict comprehensive conformational sampling and prevent the observation of slower dynamic processes that could be significant for understanding the binding cooperativity or allosteric effects [82,83,84,85]. Future studies should employ substantially longer trajectories with multiple independent replicates to improve the statistical robustness and capture rare conformational transitions. Addressing these computational limitations requires an experimental validation strategy to confirm the predicted binding interfaces and functional inhibitory effects of candidate molecules.

High-resolution structural methods are essential for validating the atomic details of predicted FWP1–stefin complexes. Techniques such as X-ray crystallography and cryo-electron microscopy (cryo-EM), complemented with small-angle X-ray scattering (SAXS) and cross-linking mass spectrometry (XL-MS), should be used to determine structural conformation [86,87,88,89]. In addition, biochemical characterization using surface plasmon resonance (SPR) [90], isothermal titration calorimetry (ITC) [91], and enzymatic kinetics assays is crucial for validating binding events, determining dissociation constants, and establishing whether inhibition follows the proposed competitive mechanism [92,93,94]. Selectivity profiling against human cathepsins, particularly cathepsins L and B, is crucial for further defining the therapeutic window and assessing potential off-target effects [95,96,97].

Experimental validation should include cell-based assays using *N. fowleri* trophozoites to demonstrate that STFA effectively suppresses FWP1 activity in a biological milieu and mitigates cytotoxicity in host cells. This cellular approach should evaluate the concentration-dependent inhibition of amoebic cytopathic effects, measure the reduction in host cell death, and assess the impact on amoebic invasion and proliferation [34,98]. Furthermore, considering the potential occurrence of compensatory mechanisms, future investigations should include long-term passaging experiments with subinhibitory STFA concentrations to monitor resistance development [99].

### 3.5. Broader Impact and Future Directions

This study highlights the value of combining AlphaFold2-Multimer modeling, MD simulations, and MM-PBSA calculations in the early stages of drug development for neglected diseases [82,100,101]. In particular, this computational pipeline successfully identified a druggable region, elucidated the mechanisms of inhibition, and provided a structural foundation for the rational design of inhibitors targeting the FWP1 protease of *N. fowleri*. The increasing accessibility of high-quality structures and the cost efficiency of virtual screening of small-molecule inhibitor libraries compared with experimental high-throughput approaches make this unifying strategy particularly advantageous for rapid target identification and inhibitor assessment in understudied pathogens that lack extensive experimental structural data [102,103].

In addition, the in silico approach described in this study may be applicable beyond *N. fowleri*, potentially benefiting studies with related pathogenic free-living amoebae, such as *Acanthamoeba* spp. and *Balamuthia mandrillaris*, which also cause fatal CNS infections and express CP enzymes [104]. Structural analyses of FWP1 orthologs could identify conserved druggable regions, paving the way for the development of broad-spectrum inhibitors and revealing species-specific adaptations that can facilitate the selective targeting of these pathogens [38,105]. Moreover, the broader relevance of other parasitic infections strengthens therapeutic strategies against other proteases and accelerates drug discovery efforts for a broader range of neglected tropical diseases [106,107].

Ultimately, this study offers a comprehensive computational framework designed to accelerate drug development against *N. fowleri* and related pathogenic amoebae. The combined approach of in silico prediction and targeted experimental validation presented here sets a promising paradigm for addressing rare but lethal infectious diseases that have historically received limited research attention. Immediate priorities include experimental validation of FWP1-STFA binding and proof-of-concept studies using *N. fowleri* infection models, as they are essential for assessing the efficacy of the proposed therapeutic strategy and supporting its clinical translation.

## 4. Materials and Methods

### 4.1. Sequence Retrieval and Database Searching

The deduced polypeptide sequences of FWP1 (FDP41_011938), FSTF (NF0067710), and NfCPI (NF0117700) were retrieved from AmoebaDB [108], a graphical and intuitive user interface that is a component of the EuPathDB project [109,110], using their respective codes (https://amoebadb.org/, accessed on 1 March 2025). The protein sequence of human STFA was retrieved from UniProt using the code P01040 (https://www.uniprot.org/, accessed on 1 March 2025). UniProt is a comprehensive database of protein information that includes current/curated polypeptide sequences, functional annotations, and cross-references [111].

### 4.2. Primary and Secondary Structure Analyses

The protein physicochemical parameters were calculated using the ProtParam tool available on the Expasy server (https://web.expasy.org/protparam/, accessed on 2 March 2025). This tool provides essential data on the physical and chemical properties of polypeptide sequences [112]. Conserved domains were identified using CD-Search on the NCBI server (https://www.ncbi.nlm.nih.gov/Structure/cdd/, accessed on 2 March 2025), which uses BLAST algorithms to quickly scan pre-calculated position-specific scoring matrices against polypeptide sequences [113,114]. Protein architecture was further confirmed using the SMART v10 tool (http://smart.embl-heidelberg.de/, accessed on 3 March 2025), which helped analyze domain organizations [115]. The secretory peptide was identified using two online predictors, SignalP 6.0 [116] and TargetP 2.0 [117], which detect protein sorting signals associated with subcellular locations (https://services.healthtech.dtu.dk/, accessed on 3 March 2025).

The protein secondary structure was predicted using PSIPRED v4.0, with default parameters, available at the UCL Department of Computer Science Workbench server (http://bioinf.cs.ucl.ac.uk/psipred/, accessed on 4 March 2025), which provides a suite of prediction tools for protein structural annotations [118]. Multiple sequence alignments were generated with Clustal Omega v1.2.4 (https://www.ebi.ac.uk/Tools/msa/clustalo/, accessed on 4 March 2025), using the HHalign algorithm under default conditions. This program applies seeded guide trees and HMM profile–profile techniques to align three or more polypeptide sequences [119].

### 4.3. Modeling of Protein Tertiary Structures

3D models of FWP1 and its heterodimeric complexes (FWP1–stefin) were generated using AlphaFold v1.3.0 and AlphaFold2-Multimer packages. AlphaFold is an artificial intelligence (AI) software that predicts the tertiary structure of proteins with atomic-level precision [103,120]. AlphaFold2-Multimer is an extension developed to predict complexes by training AlphaFold for multimeric inputs of known stoichiometry, resulting in enhanced accuracy of the predicted protein–protein interfaces while maintaining intrachain accuracy [120,121]. For the FWP1–stefin heterodimers, the complexes were modeled with a 1:1 stoichiometry. Modeling utilized three recycles with default multiple sequence alignment parameters, whereas structural templates were disabled to reduce bias from existing homologs. The structural quality of the top-ranked models generated for each complex was assessed using predicted template modeling (pTM) and interface-predicted template modeling (ipTM) scores. Models with pTM values above 0.5 and ipTM values greater than 0.8 were considered confident and high-quality complex predictions. The highest-ranked model, which displayed a consistent structure across replicates, was selected for downstream analysis.

MD simulations were performed to refine and stabilize the predicted 3D structures using the CHARMM36m force field and TIP3P water model under NPT conditions at 298 K. Each system was solvated in a cubic box, neutralized, and ionized to a final concentration of 0.15 M NaCl solution. Long-range electrostatic interactions were treated using the Particle-Mesh Ewald (PME) method, with a 12 Å cutoff for short-range non-bonded interactions and a 10 Å smoothing function. The motion equations were integrated using the r-RESPA multiple time-step scheme (updating short-range interactions at every step) and long-range electrostatics in two steps (with 2 fs integration). The temperature was controlled at 298 K using a Langevin thermostat and the pressure was maintained at 1 atm using a Nosé-Hoover barostat. Periodic boundary conditions were applied in the simulations. Prior to the production runs, the systems underwent: (1) 20,000-step minimization using the steepest-descent algorithm; (2) 0.24-ns annealing from 60 to 298 K under backbone restraints; and (3) 1-ns equilibration at 298 K with maintained restraints.

The simulations were performed using the biocomputational resources of VMD and QwikMD. VMD is a program used for the visualization and analysis of MD trajectories, with the capability of displaying animations of protein motion during simulations [122]. QwikMD is an integrated plugin that assists in the preparation, execution, and analysis by integrating two programs widely used for protein dynamics studies: VMD and NAMD [123]. Production simulations were run for 20 ns, and structural stability was evaluated by monitoring the RMSD and interaction energy profiles. Clustering analysis was performed on the final 2 ns of each trajectory to identify the most stable conformations of the FWP1–stefin heterodimers, a commonly employed approach for capturing homogeneous ensembles in MD studies [124]. Although the 20 ns timescale is a relatively short sampling window, it was sufficient to assess local stability, refine the models, and detect interaction profiles. All 3D structures were analyzed using UCSF Chimera, a user-friendly molecular visualization system [125,126].

Binding affinity values, representing the strength of protein–protein interactions, were computed using CaFE, a tool that implements the molecular mechanics Poisson–Boltzmann surface area (MM-PBSA) approach as the end-point free energy prediction method [127]. The binding energies were calculated using 100 frames from the final 2 ns of the trajectory, and a sampling method was selected to balance the computational efficiency and statistical robustness. The standard settings included a 1.4 Å probe radius, MDH boundary conditions, and an SPL2 loading scheme. The atomic radii were assigned using PARSE, and the non-polar solvation was modeled as γ·SASA (γ = 0.005 and β = 0.0). Polar solvation energies were calculated using APBS with default protein/solvent dielectric constants and an ionic strength of 0.15 M, as described previously. The binding free energy was calculated using the equation ΔG_bind_ = ΔE_MM_ + ΔG_solv_ − TΔS, where ΔE_MM_ is the molecular mechanics energy, ΔGsolv is the solvation free energy, and TΔS is the entropic contribution to the free energy. Following the usual practice for comparative MM-PBSA studies, the entropic term was omitted because its effects tend to partially cancel out when evaluating similar complexes [128,129]. Calculations were performed at 298 K, and the free energy was reported as the average and standard deviation. Notably, the ΔG MM-PBSA values were interpreted as relative binding affinities rather than absolute free energies, with more negative values indicating stronger predicted binding energies. The CTSL-STFA complex (PDB Entry ID code: 3KSE) was used as a control to validate the computational pipeline.

### 4.4. In Silico Protein–Protein Interface Interaction Analysis

Non-covalent interactions at the FWP1–stefin interfaces were systematically analyzed using two complementary methods: PLIP and DIMPLOT. For each complex, the most stable cluster from the final 2 ns of the MD trajectory was selected for the interaction analysis. PLIP (v2.3.0), a web-based tool capable of analyzing structures to detect protein-ligand and protein–protein interactions [130], was used to examine the complex conformation (https://plip-tool.biotec.tu-dresden.de/plip-web/plip/, accessed on 1 April 2025). Interaction profiling was then refined using DIMPLOT, a component of the LigPlot+ suite (v2.3), which outlines the non-covalent interactions of protein–protein interfaces at the residue level [44,45].

### 4.5. Binding Site Analysis and Druggability Evaluation

Binding hotspot identification was performed using FTMap (https://ftmap.bu.edu/, accessed on 10 September 2025). This computational server identifies binding hotspots on protein surfaces using multiple-probe molecular docking simulations [131]. The algorithm places small organic molecules as probes across the surface, followed by clustering them based on favorable interaction energies and ranking the clusters to determine the regions with the highest propensity for ligand binding. The resulting consensus sites represent potential druggable hotspots that may contribute to binding affinity and are crucial for structure-based inhibitor design. To complement the detection of hotspots, evolutionary conservation analysis was conducted using ConSurf (https://consurf.tau.ac.il/, accessed on 10 September 2025), which is a web-based tool that estimates the conservation of amino acid positions within proteins by analyzing the phylogenetic relationships between homologous sequences [132]. This method automatically collects homologous sequences, generates multiple sequence alignments, constructs phylogenetic trees, and calculates position-specific conservation scores using an empirical Bayesian algorithm. These scores range from 1 (highly variable) to 9 (highly conserved), with functionally and structurally relevant residues showing high conservation values. This analysis was performed on FWP1 and human CTSL to identify conserved residues and species-specific variations that could contribute to selective inhibitor binding.

The ligand-binding site of FWP1 was investigated using an advanced extension of the established CASTp algorithm [133], CASTpFold (https://cfold.bme.uic.edu/castpfold/, accessed on 9 September 2025), which offers improved accuracy and comprehensive characterization of protein topographical features. While preserving the functions of its predecessor, CASTpFold enhances the analysis with a detailed topographical–topological quantification of both predicted models and experimental structures [134]. Building on rigorous computational principles, it identifies pockets and cavities with high precision, delineates the boundary between solvent-accessible regions and enclosed pockets, and ensures rotationally invariant orientation-independent measurements without reliance on grid-based discretization. The FWP1 pocket was identified using a probe radius of 1.4 Å. Druggability was evaluated using PockDrug (http://pockdrug.rpbs.univ-paris-diderot.fr/, accessed on 5 April 2025) and DoGSiteScorer, available on the Proteins Plus server (https://proteins.plus/, accessed on 10 September 2025). PockDrug utilizes a robust algorithm designed to address uncertainties in pocket boundaries. It predicts druggability models for both pockets guided by ligand proximity and not guided by ligand data [135,136]. DoGSiteScorer uses a grid-based Difference of Gaussian filter to detect potential binding pockets and provides a druggability score derived from a support vector machine (SVM) trained on a linear combination of pocket descriptors (volume, hydrophobicity, and enclosure). For both methods, druggability probabilities >0.5 were indicative of a druggable site, consistent with standard practice [137]. All analyses were performed using the apo FWP1 structure.

## 5. Conclusions

FWP1, a papain-like cysteine protease secreted by *N. fowleri*, is a reliable therapeutic target. Computational modeling, molecular dynamics, and binding energy calculations revealed that stefins could form stable complexes with FWP1 and identified the crucial interaction motifs. Druggability analysis revealed that a region within this amoebic protease possesses topological and physicochemical properties typical of high-affinity ligand-binding sites. Notably, human STFA exhibited a strong binding affinity and favorable interaction profile, highlighting its potential as a biopharmaceutical lead. Overall, this study provides a framework for integrating stefins into structure-based drug discovery approaches aimed at targeting FWP1 for therapeutic interventions in *N. fowleri* infection.

Future investigations will focus on the recombinant cloning and expression of FWP1, followed by activity assays using fluorogenic substrates in the presence and absence of stefins. Comprehensive enzyme kinetic analyses, including the determination of inhibitory constants (K_i_) and association and dissociation rates (k_on_ and k_off_), will be conducted to quantify the inhibitory efficiency. In addition, structural biophysics methods will be employed to resolve the details of inhibitor–protease interactions at atomic-level resolution. Finally, translational studies will advance to in vivo validation using an animal model of primary amoebic meningoencephalitis, with further pharmacokinetic analyses of candidate stefins performed in cerebrospinal fluid.

## Figures and Tables

**Table 1:** Binding free energy (ΔG) and equilibrium dissociation constant (K_d_) of stable heterodimeric complexes.

Protease-Stefin Complex ^1^	MD Simulation ^2^		Experimental ^3^ K_d_ (M)
	ΔG (kcal/mol)	K_d_ (M)	
FWP1-FSTF	−23.6 ± 9.8	4.7 × 10^−18^	-
FWP1-NfCPI	−17.9 ± 9.5	7.5 × 10^−14^	-
FWP1-STFA	−22.2 ± 9.1	5.3 × 10^−17^	-
CTSL-STFA	−16.2 ± 8.3	1.4 × 10^−12^	≤1 × 10^−11^

^1^ Abbreviations as specified in Figure 3. ^2^ Parameters calculated from the most stable clusters using the MM-PBSA method. ΔG values were converted to K_d_ using the formula ΔG = RT · ln Kd, where R = 1.987 cal mol^−1^ K^−1^ and T = 298 K. ^3^ K_d_ value reported for the human CTSL-STFA complex [43].

## Data Availability

All data generated in this study are described here or reported as Supplementary Materials. Please contact the corresponding author for any additional inquiries.

## Supplementary Materials

The following supporting information can be downloaded at: https://www.mdpi.com/article/10.3390/ph18111606/s1. Figure S1. Ribbon representation of the best 3D model of Fowlerpain-1. Figure S2. Structural analysis of the best 3D model of Fowlerpain-1. Figure S3. Best 3D models of the FWP1–stefin complexes. Figure S4. Multiple sequence alignment of fowlerstefin (FSTF), *N. fowleri* cysteine protease inhibitor (NfCPI), and human stefin A (STFA). Figure S5. FTMap interaction plots of FWP1. Table S1. Receptor and ligand residues with conserved non-covalent interactions at the FWP1–stefin complex interfaces. Table S2. Comparative residue conservation analysis of FWP1 and CTSL based on ConSurf profiling.

## Abbreviations

The following abbreviations are used in this manuscript:

CP	Cysteine protease
CPI	Cysteine protease inhibitor
CTSL	Cathepsin L
FSTF	Fowlerstefin
FWP1	Fowlerpain-1
Nf	*Naegleria fowleri*
PAM	Primary amoebic meningoencephalitis
STFA	Stefin A
