# Supplementary material for: Structure-Based Insights into Stefin-Mediated Targeting of Fowlerpain-1: Towards Novel Therapeutics for Naegleria fowleri Infections
Source: Pharmaceuticals (Basel). 2025 Oct 23;18(11):1606. doi: 10.3390/ph18111606 (PMC12655743; doi:10.3390/ph18111606)
Supplement: Supplementary file 1 [file pharmaceuticals-18-01606-s001.zip › pharmaceuticals-3840697-supplementary.pdf]

Pharmaceuticals

Supplementary Material

Structure-Based Insights into Stefin-Mediated Targeting of Fowlerpain-1: Towards Novel Therapeutics for *Naegleria fowleri* Infections

Pablo A. Madero-Ayala, Rosa E. Mares-Alejandre, Patricia L.A. Muñoz-Muñoz, Samuel G. Meléndez-López, and Marco A. Ramos-Ibarra*

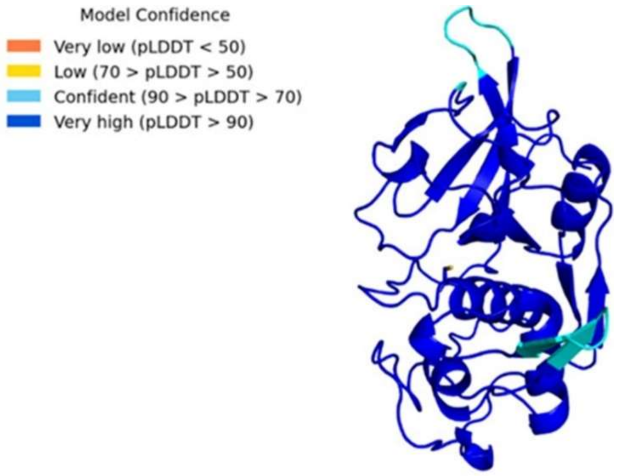

Figure S1. Ribbon representation of the best 3D model of Fowlerpain-1. The colors used for sequence annotation according to the confidence level are shown on the upper left side (default settings). The relative position of the catalytic Cys residue is represented by the colored sticks.

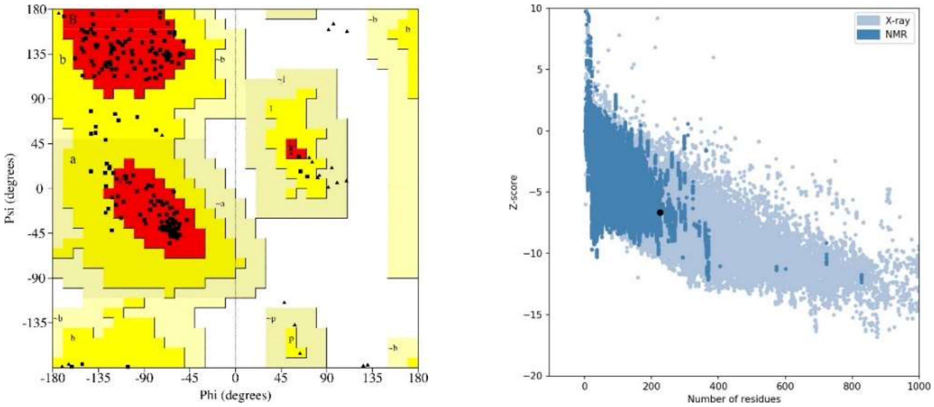

A **B**

Figure S2. Structural analysis of the best 3D model of Fowlerpain-1. Ramachandran (**A**) and ProSA (**B**) plots.

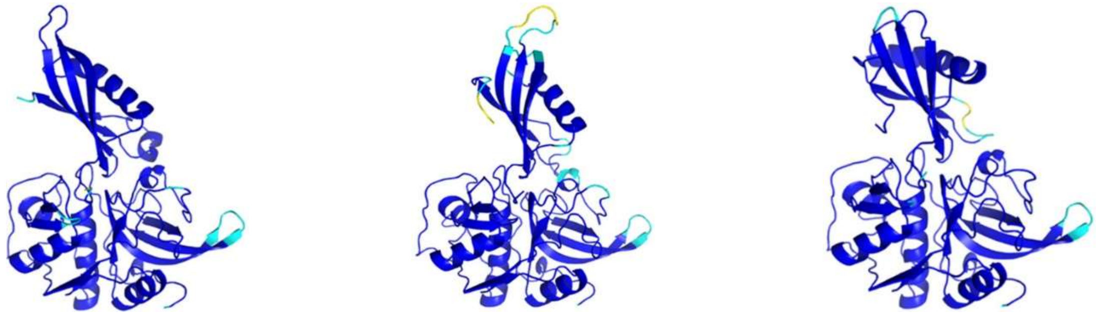

A B C

Figure S3. Best 3D models of the FWP1-stefin complexes. Ribbon representation of heterodimers formed by Fowlerpain-1 in complex with fowlerstefin (**A**), NfCPI (**B**), and human stefin A (**C**). The color codes used for sequence annotation according to the confidence level are presented in Figure S1.

```
FSTF         1 ------------MKKIILVALFLIVGLLFLANQSPSEASVVPGGKSLERNK-QTIKALGEFLNSKLSTTDYASYSVEKVI 67
NfCPI        1 MMINNLSAPAAATTLIFILALLITFS---NILALPIATNVIPGGRQPISNQ-TLIEELTTFIPKASS---NGVCRVTRVL 73
STFA         1 ---------------------------------------MIPGGLSEAKPATPEIQEIVDKVKPQLEEKTNETYGKLEAV 41
Conserved                                               ***          *

FSTF        68 AVERQVVAGVNYFVRAKIQAHGSQH---AKIIEAKIFEPLPYMIKQGA-EPYKLVSVSERR------- 124
NfCPI       74 SAQQQVVAGTIYYLVVEVSTSCDATSSQTFCEKLKIFRPLVTRS---V-ENPQLQLQKEQRVDCNTPA 137
STFA        42 QYKTQVVAGTNYYIKVRAGDN--------KYMHLKVFKSLPGQNEDLVLTGYQVDKNKDDELTGF---  98
Conserved          *****  *                      * *  *
```

Figure S4. Multiple sequence alignment of fowlerstefin (FSTF), *N. fowleri* cysteine protease inhibitor (NfCPI), and human stefin A (STFA). All identical and similar residues are shaded gray, whereas those conserved among the three stefins are indicated by asterisks (*).

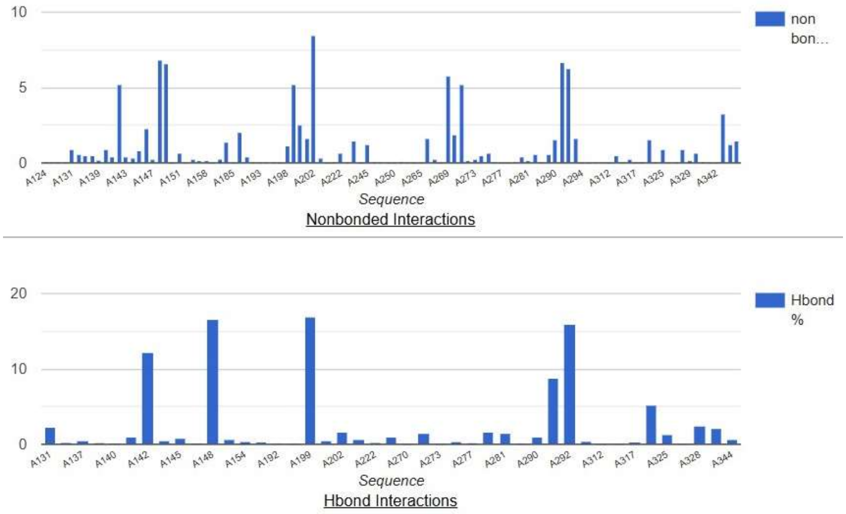

Figure S5. FTMap interaction plots of FWP1. Predicted ligand-binding hotspots on FWP1 were identified using FTMap (https://ftmap.bu.edu/). The plots highlight the non-bonded contacts and hydrogen-bond interactions between the FWP1 residues and probe molecules, delineating the key physicochemical anchors that characterize the predicted druggable region.

Table S1. Receptor and ligand residues with conserved non-covalent interactions at the FWP1-stefin complex interfaces.

Receptor		Ligands [1]					
Fowlerpain-1 (FWP1)		Fowlerstefin		NfCPI		Human STFA	
143	Gly	75	Ala	81	Ala	49	Ala
144	Ala	74	Val	80	Val	48	Val
145	Cys	74	Val	80	Val	48	Val
146	Gly	74	Val	ND	ND	48	Val
148	Cys	30	Pro	39	Pro	3	Pro
148	Cys	31	Gly	40	Gly	4	Gly
196	Cys	72	Gln	78	Gln	46	Gln
198	Gly	31	Gly	40	Gly	4	Gly
199	Gly	30	Pro	39	Pro	3	Pro
200	Leu	30	Pro	39	Pro	3	Pro
266	Ala	30	Pro	39	Pro	3	Pro
291	Asp	30	Pro	39	Pro	3	Pro
291	Asp	31	Gly	40	Gly	4	Gly
291	Asp	73	Val	79	Val	47	Val
292	His	30	Pro	39	Pro	3	Pro
319	Trp	74	Val	79	Val	48	Val
319	Trp	75	Ala	81	Ala	49	Ala

[1] Ligand residues with hydrophobic interactions or hydrogen bonding properties are lightly shaded yellow and blue, respectively. Abbreviations: NfCPI, *N. fowleri* cysteine protease inhibitor; STFA, stefin A; ND, not detected.

Table S2. Comparative residue conservation analysis of FWP1 and CTSL based on ConSurf profiling.

Fowlepain 1 (FWP1)			Cathepsin L (CTSL)		
Group	SeqNo	ConSurf [1]	Group	SeqNo	ConSurf [1]
GLN	142	9	GLN	132	9
GLY	143	6	GLY	133	6
ALA	144	3	GLN	134	4
CYS	145	9	CYS	135	9
GLY	146	8	GLY	136	9
CYS	148	8	CYS	138	8
CYS	196	9	CYS	178	9
GLY	198	8	GLY	180	8
GLY	199	9	GLY	181	9
LEU	200	3	LEU	182	1
ALA	266	5	ALA	248	5
ASP	291	7	ASP	275	7
HIS	292	9	HIS	276	9
TRP	319	9	TRP	302	8

[1] Residue-level conservation profiles highlighting the catalytic residues, structural stabilizers, and divergent positions within the active-site region. Highly conserved residues (score 9) denote shared evolutionary and functional constraints, whereas variable residues identify potential sites for the design of selective inhibitors.
